# A Rat Immobilization Model Based on Cage Volume Reduction: A Physiological Model for Bed Rest?

**DOI:** 10.3389/fphys.2017.00184

**Published:** 2017-03-29

**Authors:** Enrica Marmonti, Sílvia Busquets, Míriam Toledo, Marina Ricci, Marc Beltrà, Victòria Gudiño, Francesc Oliva, José M. López-Pedrosa, Manuel Manzano, Ricardo Rueda, Francisco J. López-Soriano, Josep M. Argilés

**Affiliations:** ^1^Cancer Research Group, Facultat de Biologia, Departament de Bioquímica i Biomedicina Molecular, Universitat de BarcelonaBarcelona, Spain; ^2^Institut de Biomedicina de la Universitat de BarcelonaBarcelona, Spain; ^3^Facultat de Biologia, Departament de Genètica, Microbiologia i Estadística, Universitat de BarcelonaBarcelona, Spain; ^4^Abbott NutritionGranada, Spain

**Keywords:** disuse-induced atrophy, physical inactivity, body composition, muscle performance, bone density, corticosteroids

## Abstract

Bed rest has been an established treatment in the past prescribed for critically illness or convalescing patients, in order to preserve their body metabolic resource, to prevent serious complications and to support their rapid path to recovery. However, it has been reported that prolonged bed rest can have detrimental consequences that may delay or prevent the recovery from clinical illness. In order to study disuse-induced changes in muscle and bone, as observed during prolonged bed rest in humans, an innovative new model of muscle disuse for rodents is presented. Basically, the animals are confined to a reduced space designed to restrict their locomotion movements and allow them to drink and eat easily, without generating physical stress. The animals were immobilized for either 7, 14, or 28 days. The immobilization procedure induced a significant decrease of food intake, both at 14 and 28 days of immobilization. The reduced food intake was not a consequence of a stress condition induced by the model since plasma corticosterone levels –an indicator of a stress response– were not altered following the immobilization period. The animals showed a significant decrease in soleus muscle mass, grip force and cross-sectional area (a measure of fiber size), together with a decrease in bone mineral density. The present model may potentially serve to investigate the effects of bed-rest in pathological states characterized by a catabolic condition, such as diabetes or cancer.

## Introduction

Bed rest has been an established treatment in the past prescribed for critically illness or convalescing patients, in order to preserve their body metabolic resource, to prevent serious complications and to support their rapid path to recovery. However, in the past 50 years numerous scientific studies have reported that physical inactivity can exert negative effects to the entire system (Corcoran, 1991). Prolonged bed rest can have detrimental consequences that may delay or prevent the recovery from clinical illness including insulin resistance, thromboembolic disease, degenerative joint disease, disuse osteoporosis, respiratory, and musculoskeletal complications (Dittmer and Teasell, 1993). In addition to different pathological catabolic conditions –such as trauma, cancer and sepsis- healthy aging (sarcopenia) and disuse (bed rest, microgravity) are also associated with muscle atrophy and wasting. Particularly, disuse-induced muscle atrophy is a catabolic condition commonly manifested in patients enforced to periods of muscle inactivity often associated with situations such as hindlimb immobilization, prolonged bed rest due to aging or the recovery from injuries, sepsis, and illnesses or weightlessness, as occurs in spaceflight (Musacchia et al., 1988). It is important to point out that unlike the muscle wasting caused by some disease states, disuse atrophy is initiated by a reduction in muscle contractile activity and muscle tension, rather than by inflammatory cytokines (Jackman and Kandarian, 2004; Chopard et al., 2009).

Physical inactivity is often achieved in humans using the bed rest or the unilateral limb suspension model, whereas the most frequently used models in rodents are denervation, casting immobilization or tail suspension (Morey-Holton and Globus, 2002; Frimel et al., 2005; Midrio, 2006). Each procedure has specific advantages and strengths which promote its use, as well as disadvantages which limit data interpretation and differ each other in term of degree of reproduced inactivity (Machida and Booth, 2004) and distinct protein degradation profiles induced (Bialek et al., 2011).

In this regard, we have designed a non-invasive, practical and low-cost model for rodents that better mimics human's reduced daily ambulatory motions, reproducing the effects of acute transitions from high to low levels of physical activity, and that better simulates the metabolic state of a patients in bed rest condition. Although immobilization through the restriction of locomotion is not a novel approach in animal experimentation—it has already been applied in biobehavioural research, particularly for the study of the stress response (Hauger et al., 1988; Wood et al., 2003; Buynitsky and Mostofsky, 2009)—its application on the study of muscle atrophy is completely new. Bearing all this in mind, the object of the present study was to design and test an immobilization method for rodents based on reduction of cage volume, to mimic the situation encountered in humans in bed rest. In particular, this initial study concentrates on the metabolic, functional, and morphometric characterization of the temporal progression of changes in muscle and bone mass, together with changes in glucose metabolism.

## Materials and methods

### Setting and procedure

**Figure 1:**
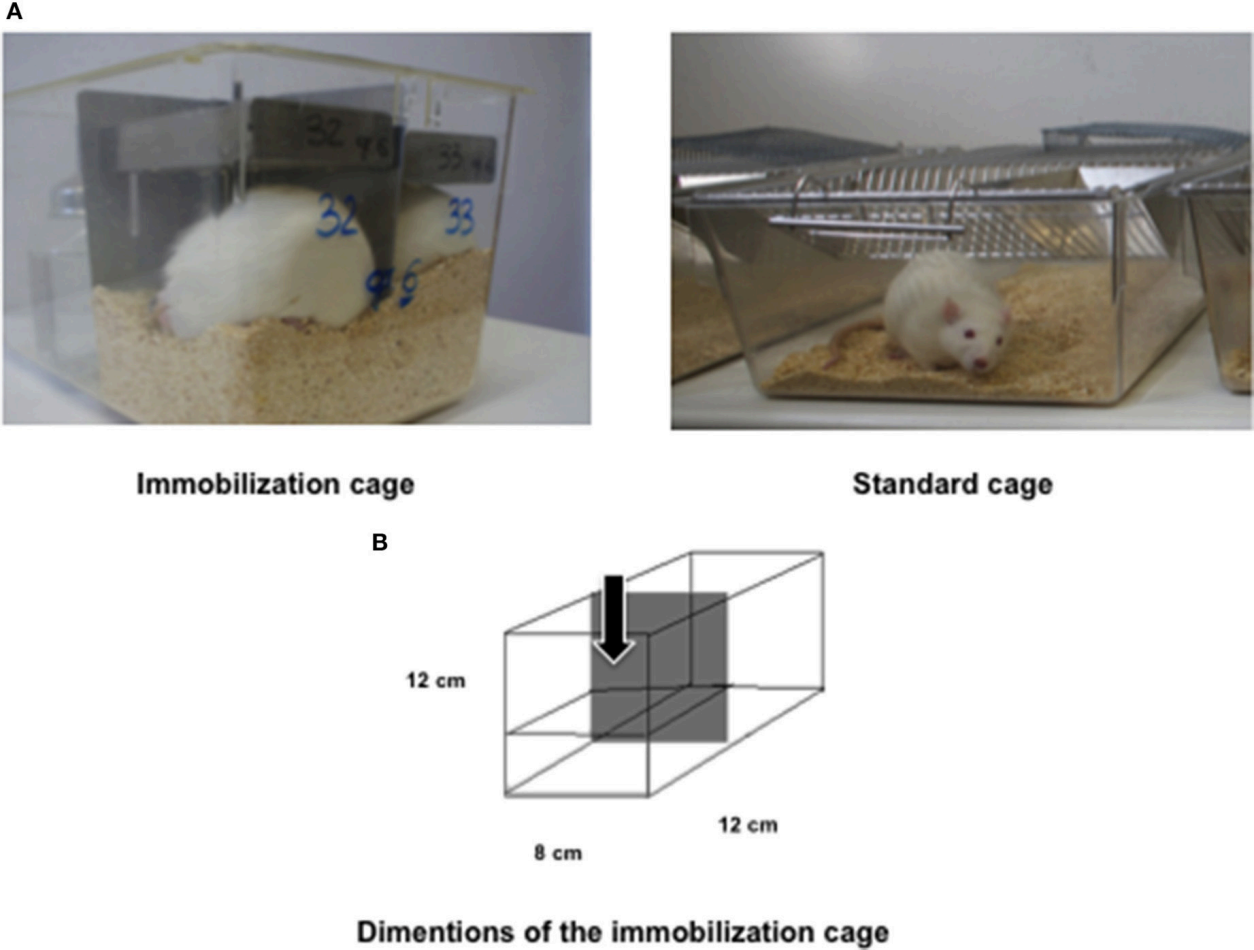
**Immobilization model**. **(A)** Representative pictures of immobilized rats and control rat in a standard cage. Each immobilization cage is able to host two animals and it is equipped (in its upper part) with a grating to dispose food and water. **(B)** Schematic representation of the immobilization tested model. The cage had a reduction in volume of 80% in relation with control standard cage. The arrow indicated the housing area of a single rat.

**Figure 2:**
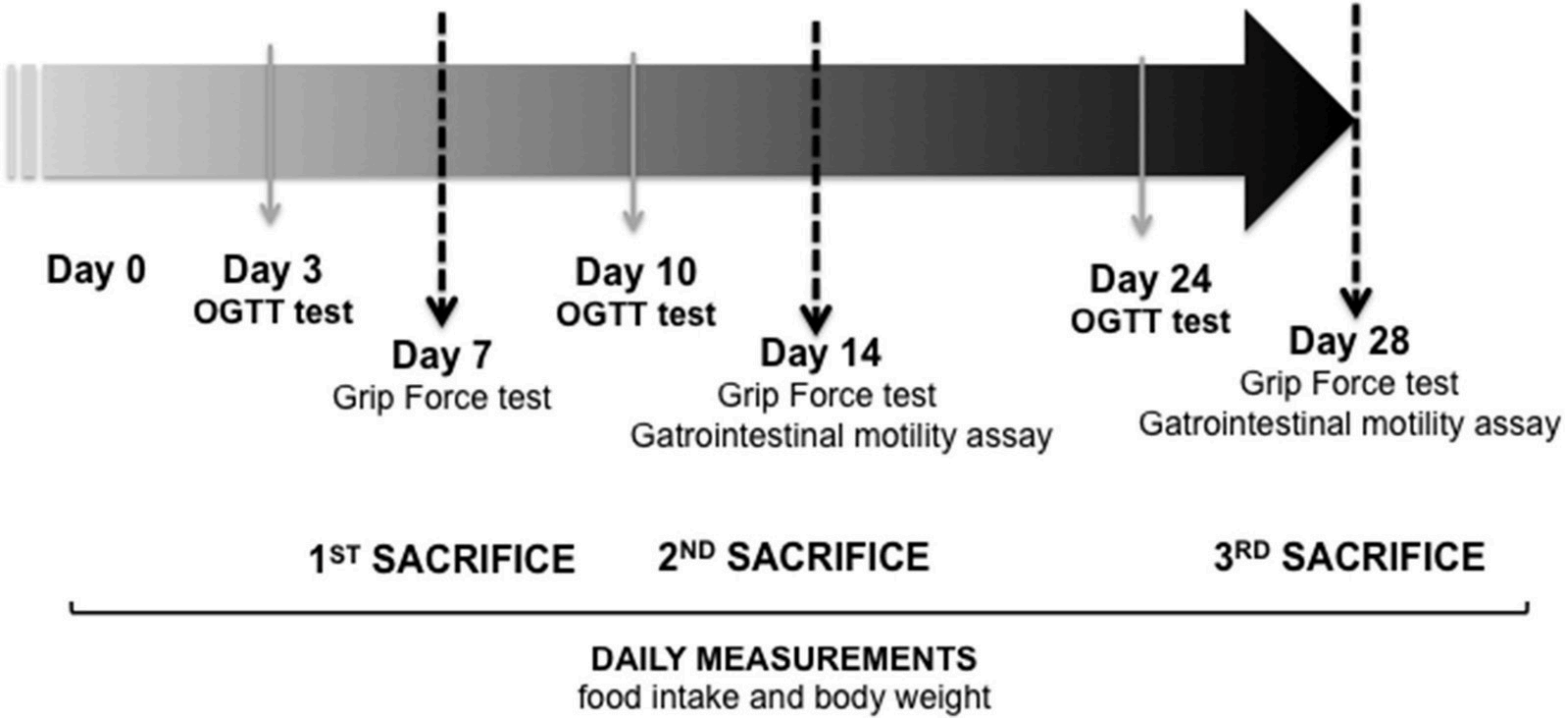
**Timeline of the experimental plan**. Day 7: CONTROL (*n* = 5) and immobilized group (IMMO) (*n* = 6); day 14: CONTROL (*n* = 9) and immobilized group (IMMO) (*n* = 12); day 28: CONTROL (*n* = 5) and IMMO (*n* = 6). OGTT: Oral glucose tolerance test.

Male Wistar rats (11 weeks-old) were housed individually and maintained on a regular light-dark cycle (light on from 08:00 a.m. to 08:00 p.m.) controlled temperature (22°C) and humidity (45%) and they had free access to water and food (AIN93M diet) (Reeves et al., 1993). They were divided into two groups: control (standard cage, *n* = 5) and immobilized (IMMO, reduced volume cage, 7–28 days *n* = 9; 14 days *n* = 12). The immobilized animals were kept for 7, 14, and 28 consecutive days in a reduced volume cage (*Tecniplast 2150*), the space being restricted to 12 × 12 × 8 cm (approximately an 80% reduction in the total standard cage volume) (Figure 1). Body weight, food and water intake were recorded daily. Rats were sacrificed at day 7, 14, and 28. Prior to sacrifice rats were weighed and anesthetized (3:1 mixture of ketamine (Imalgene®) and xylazine (Rompun®). Blood was collected from the aorta and post-prandial plasma separated by centrifugation at 3,500 g for 10 min at 4°C and stored at −80°C. Muscles and other tissues were rapidly excised, weighed and frozen in nitrogen liquid. All tissues were stored at − 80°C until analysis. For a better comprehension, a timeline of the experimental plan is represented in Figure 2. All animal manipulations were made in accordance with the European Community guidelines for the use of laboratory animals. They were cared for in compliance with the *Policy on Humane Care and Use of Laboratory Animals* (ILAR 2011). The Bioethical Committee of the University of Barcelona approved the experimental protocol.

### Outcome parameters

#### Corticosteroids

At day 1, 4, 8, and 14, blood samples were collected from the distal extreme of the tail at the same time (3:00 p.m.) in order to avoid the “screen” effect of the circadian rhythm on the response to stress (Smith, 2012). The number of animals used to measure the corticosteroid levels were the following: day 1: CONTROL *n* = 4 and immobilized group (IMMO) *n* = 5; day 4: CONTROL *n* = 5 and IMMO *n* = 5; day 8: CONTROL *n* = 5 and IMMO *n* = 4; day 14: CONTROL *n* = 4 and IMMO *n* = 4. Plasma corticosterone levels were quantified by ELISA kit (*Arbor assays, Chicago, USA)*.

#### Oral glucose tolerance test (OGTT)

The OGTT was performed 4 days prior to sacrifice in order to minimize the influence of the fasting on the final body weight, body composition and physical activity tested the day of sacrifice. Animals were fasted overnight (12 h) and blood was collected in heparinised wells from the distal extreme of the tail, prior to the glucose solution administration, to assess the fasting levels of glucose and insulin (baseline time). To reduce an infection, a topical germicide (Betadine® solution) was applied to the tail following blood collection. Blood collection was obtained 15, 30, 60, and 120 min after the glucose solution administration (2 g/kg rat) by gavage. Glucose levels were measured by the Glucometer (*Accutrend, GCT, Mannheim, Germany, Roche*).

#### Grip strength

Skeletal muscle force in rats was quantified by the grip-strength test once a week. The grip strength device (*Panlab-Harvard Apparatus, Spain*) comprised a pull bar connected to an isometric force transducer (dynamometer). Basically, the apparatus was positioned horizontally and the rats were held by the tail and lowered toward the device. The animals were allowed to grasp the pull bar by their forelimbs and were then pulled backwards in the horizontal plane. The force applied to the bar just before the animals lost grip was recorded as the peak tension. At least three measurements were taken per rat on both baseline and test days, and the results were averaged for analysis. This force was measured in grams/grams initial body weight (Toledo et al., 2011).

#### Gastrointestinal motility

Gastrointestinal motility in rats was tested by a method described (Arbós et al., 1993). An oral glucose load (4 mmol) containing 2 μCi of [^3^H]inulin was administrated to each rat 2 h before the sacrifice. The gastrointestinal tract was extracted and divided into stomach and intestine with their contents and they were processed for [^3^H]-scintillation counting. The intestine was divided into six equivalent segments (duodenal to colon: I1–I6) and the amount of labeled retained calculated for each of them. They were mixed with 3% (w/v) perchloric acid and homogenized in a Waring Blender. After centrifugation, 5 mL of the supernatant were neutralized with potassium hydroxide 30% and then centrifuged at 1,000 g for 5 min to accelerate the ${\text{ClO}}_{4}^{-}$ precipitation under form of KClO4. Finally, 5 mL of the neutralized supernatant were added to 10 mL of scintillation fluid for the measurement of total radioactivity.

#### Fiber cross sectional area

During the sacrifice, the soleus muscle was rapidly excised from each limb, and quickly frozen in liquid-nitrogen cooled isopentane, maintaining the correct orientation to allow cross section. Ten micrometers of transverse sections from the mid-belly of the muscles were cut on a cryostat at −20°C. The slides obtained were stained by haematoxylin-eosin staining protocol, mounted with permount mounting media (*Fisher, United States*) and photographed at 10X magnification. Fiber cross-sectional area (CSA) was determined on randomly chosen 100 individual fibers per animal by the *Image J software* and expressed in pixels (Abramoff et al., 2004). Photo magnification and resolution were maintained fixed within each experiment.

#### Body composition analysis

Body composition was determined *post-mortem* in the all body of the animals, excluding tissues used in other measures (muscles, organs, and blood), by quantitative magnetic resonance (QMR) by means of an Echo MRI-100 rodent whole body composition analyser (Echo Medical Systems, Houston, Texas, USA) (Nixon et al., 2010).

#### Bone mineral density analysis

Bone mineral density was measured *post-mortem* in tibia, femur, lumbar vertebras (LV 2-5), forearm, and humerus by peripheral Dual-energy X-ray Absorptiometry (pDEXA) analysis (Norland Corp., Fort Atkinson, WI, USA) (Griffin et al., 1993).

### Statistical analysis

To summarize and describe the results, average (arithmetic mean), and standard error of the mean (SEM) were calculated for each studied variable. Intergroup differences were evaluated using analysis of variance (ANOVA) and linear mixed models. *Post hoc* pairwise comparisons (Duncan test) were performed when appropriated. In order to assess the validity of the ANOVA results, the normality of data and homogeneity of variances were check for each variable. All the statistical analysis was performed using SPSS (version 21).

## Results

In order to study disuse-induced changes in muscle and bone, as observed during prolonged bed rest in humans, we have designed a new model of muscle disuse for rodents. The immobilization device is depicted in Figure 1. Basically, the animals are confined to a reduced space not permitting displacement (but allowing them to drink and eat in an easy way similar to a bed rest condition) for 7, 14, and 28 consecutive days (Figure 2). However, it has to be pointed out that, in terms of lifespan, the immobilization periods used here are far longer than those previously used in human studies. Although bed rest is a unique model to investigate mechanisms of underlying defects induced by physical inactivity in healthy subjects, it is important to remember that bed rest induces a level of physical inactivity likely different (quantitatively and qualitatively) from that observed under other conditions.

The average daily food intake was decreased due to the immobilization procedure without inducing variations in the body weight of the animals (Table 1). The animals consumed a reduced amount of food to maintain the energetic balance. However, quantitative magnetic resonance data, shown in Table 1, do not evidence changes of the lean and fat mass composition after the immobilization period. No differences were observed in plasma corticosteroid levels between immobilized and non-immobilized animals (Table 2).

**Table 1:** **Body weight gain, food intake, energetic efficiency, and body composition in immobilized Wistar rats**.

**Parameters**	**7 DAYS**		**14 DAYS**		**28 DAYS**		**ANOVA**		
	**CONTROL**	**IMMO**	**CONTROL**	**IMMO**	**CONTROL**	**IMMO**	***I***	***T***	**IxT**
(FBW-IBW)	6.2 ± 3.8(5)	9.2 ± 2.3(6)	36.7 ± 4.5(9)	22.3 ± 4.8(12)	43.4 ± 9.1(5)	43.4 ± 5.1(6)	ns	0.001	ns
DAILY FOOD INTAKE	7.4 ± 0.4(5)	6.9 ± 0.2(6)	6.1 ± 0.1(9)	5.5 ± 0.2(12)	5.3 ± 0.3(5)	5.0 ± 0.2(6)	0.015	0.001	ns
ENERGETIC EFFICIENCY	4.1 ± 2.7(5)	7.0 ± 1.8(6)	13.6 ± 1.5(9)	9.0 ± 1.9(12)	9.1 ± 1.5(5)	9.9 ± 0.9(6)	ns	0.016	ns
% FAT MASS	10.3 ± 0.7(5)	10.0 ± 0.3(6)	12.2 ± 0.4(9)	11.8 ± 0.4(12)	12.2 ± 0.8(5)	11.7 ± 0.8(6)	ns	0.004	ns
% LEAN MASS	85.0 ± 1.0(5)	85.4 ± 0.6(6)	83.0 ± 0.6(9)	83.0 ± 0.4(12)	82.6 ± 0.7(5)	83.9 ± 0.7(6)	ns	0.030	ns

*Data are summarized as mean ± SEM, number of animals (replicates) indicated in parentheses for control (CONTROL) and immobilized (IMMO) groups. IBW, initial body weight, FBW, final body weight (in grams). The formula used to calculate the ENERGETIC EFFICIENCY was [(FBW-IBW)/food ingested] × 100, ingested food in grams. The formula used to calculate the DAILY FOOD INTAKE was (food ingested per day within the period/IBW) × 100, food intake are expressed as grams per 100 grams of initial body weight. Upper body composition (FAT and LEAN MASS percentage) was measured by Magnetic Resonance Imaging analysis. Statistical significant differences were evaluated by a full factorial two-way ANOVA (fixed factors: I, immobilization, T, Time; IxT denotes the interaction term), p-values are shown in the three columns on the right (ns, non-significant differences, which implies a p-value equal to or greater than 0.05)*.

**Table 2:** **Plasmatic corticosteroid levels and adrenal glands weight**.

	**CONTROL**	**IMMO**
**CORTICOSTEROID LEVELS (ng/mL)**		
Day 1	335 ± 57 (4)	242 ± 33 (5)
Day 4	212 ± 67 (5)	271 ± 66 (5)
Day 8	314 ± 48 (5)	213 ± 20 (4)
Day 14	190 ± 61 (4)	384 ± 74 (4)
**ADRENAL GLANDS (mg/100 g IBW)**		
Day 7	27.9 ± 2.7 (4)	24.0 ± 2.7 (6)
Day 14	30.2 ± 0.9 (9)	27.6 ± 1.0 (9)

*Corticosteroid levels (ng/mL) are expressed as mean ± SEM for the number of animals indicated in parentheses for control (CONTROL) and immobilized (IMMO) groups. Adrenal glands weights are expressed as milligrams per 100 grams of initial body weight. Data were analyzed by a Repeated Measures Linear Mixed Model, being time (T) the repeated measures factor and immobilization (I) a fixed-effects factor. Restricted Maximum Likelihood (REML) method was used to fit the mixed model. According to the values of Akaike Information Criterion (AIC) and Schwarz Bayesian Information Criterion (BIC), identity scaling was finally chosen as the covariance matrix structure. For corticosteroid levels: no significant differences were detected over time (T, p = 0.806) or for the immobilization factor (I, p = 0.697), neither for interaction (IxT, p = 0.052). For adrenal glands: Statistical significance was tested by a full factorial two-way ANOVA (fixed factors: immobilization (I) (p = 0.080), Time (T) (p = 0.516); interaction (IxT) (p = 0.990)*.

**Figure 3:**
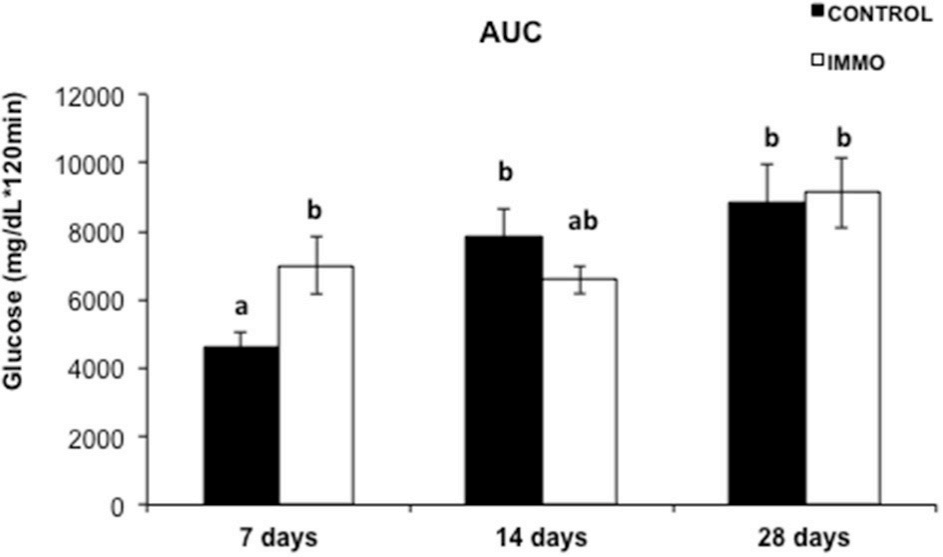
**Area under the curve (AUC) of plasma incremental glycaemia values in immobilized Wistar rats**. Each bar and segment represents mean values ± SEM for CONTROL (*n* = 5) and immobilized groups (IMMO) (*n* = 6). Statistical significant differences were evaluated by a full factorial two-way ANOVA (fixed factors: I = immobilization, T = Time; IxT denotes the interaction term): I, *p* = ns; T, *p* = 0.033; IxT, *p* = 0.021. *Post-hoc* pairwise comparisons were performed (Duncan method); different letters (a and b) indicate significant differences between the experimental groups.

We analyzed the effects of the proposed disuse model on glucose metabolism. Unexpectedly, a significantly variation on glucose tolerance was observed in the rats only after 7 days of immobilization (Figure 3).

Concerning muscle weights, immobilization resulted in a significant decrease in soleus weight (−7.3, 10.5, and 13.2% for 7-, 14- and 28 days-following immobilization, respectively) (Table 3). In addition, heart weight was also decreased after 7 and 14 days of immobilization (Table 3). In order to assess if the muscle wasting induced by our immobilization model was translated in an altered muscle performance, we measured grip force of the fore limbs of the animals. The data presented in Table 3 showed significant decrease of this parameter after 7 and 14 days of physical inactivity. The results presented in Table 4 clearly showed that physical inactivity significantly decreased bone mineral density in the vertebrae (LV-25) of the immobilized animals.

**Table 3:** **Muscle weights and grip force in immobilized Wistar rats**.

	**7 DAYS**		**14 DAYS**		**28 DAYS**		**ANOVA**		
	**CONTROL**	**IMMO**	**CONTROL**	**IMMO**	**CONTROL**	**IMMO**	***I***	***T***	**IXT**
GSN	597.6 ± 16.1(5)	604.1 ± 8.2(6)	592.2 ± 14.7(9)	607.7 ± 9.7(12)	674.4 ± 8.0(5)	659.0 ± 16.6(6)	ns	0.001	ns
EDL	47.1 ± 2.0(5)	48.4 ± 1.3(6)	45.6 ± 1.3(9)	47.2 ± 0.7(12)	49.2 ± 0.3(5)	48.6 ± 1.9(6)	ns	ns	ns
TIB	184.6 ± 10.2(5)	193.6 ± 2.3(6)	194.0 ± 5.1(9)	195.9 ± 3.1(12)	207.4 ± 5.7(5)	207.5 ± 6.3(6)	ns	0.012	ns
SOL	43.7 ± 2.9(5)	40.5 ± 0.9(6)	42.7 ± 1.5(8)	38.2 ± 0.5(12)	48.2 ± 2.1(5)	41.8 ± 0.6(6)	0.001	0.004	ns
HEART	264.8 ± 7.8(5)*b*	244.3 ± 3.0(6)*a*	276.3 ± 3.8(9)*b*	240.9 ± 7.2(12)*a*	266.7 ± 5.5(5)*b*	269.3 ± 7.7(6)*b*	0.004	ns	0.026
GRIP FORCE	3.8 ± 0.1(5)	3.3 ± 0.1(6)	3.7 ± 0.1(8)	3.5 ± 0.1(11)	4.0 ± 0.2(5)	3.8 ± 0.2(6)	0.005	0.029	ns

*Mean ± SEM for the number of animals indicated in parentheses are shown for every period for control (CONTROL) and immobilized (IMMO) groups. Muscle weights are expressed as milligrams per 100 grams of initial body weight. Statistical significance was tested by a full factorial two-way ANOVA (fixed factors: I, immobilization, T, Time; IxT denotes the interaction term), p-values are shown in the three columns on the right (ns, non-significant differences, which implies a p-value equal to or greater than 0.05). It has been detected a significant interaction term in the model for the heart, so that pairwise comparisons were performed (Duncan method); different letters indicate significant differences between the experimental groups. GSN, gastrocnemius, EDL, extensor digitorum longus, TIB, tibialis, SOL: soleus. Muscle force of the animal was tested once a week and was expressed as g/g initial body weight*.

**Table 4:** **Bone Mineral density (BMD) of immobilized Wistar rats**.

	**7 DAYS**		**14 DAYS**		**28 DAYS**		**ANOVA**		
**BONE**	**CONTROL**	**IMMO**	**CONTROL**	**IMMO**	**CONTROL**	**IMMO**	***I***	***T***	**IXT**
LV-25	175.1 ± 3.9 (5)	173.4 ± 2.6 (6)	183.5 ± 2.4 (9)	172.7 ± 2.2 (12)	189.9 ± 3.5 (5)	180.1 ± 4.2 (6)	0.006	0.011	ns
HUMERUS	159.8 ± 2.1 (5)	162.8 ± 2.2 (6)	169.6 ± 2.9 (9)	165.2 ± 2.0 (12)	175.3 ± 2.0 (5)	175.6 ± 2.6 (6)	ns	0.001	ns
FOREARM	154.9 ± 2.3 (5)	161.8 ± 2.5 (6)	162.2 ± 4.7 (9)	161.0 ± 1.9 (12)	165.9 ± 4.6 (5)	166.9 ± 3.0 (6)	ns	ns	ns
TIBIA	150.7 ± 0.9 (5)	150.6 ± 0.8 (6)	159.2 ± 0.9 (8)	152.9 ± 1.9 (12)	165.6 ± 1.8 (5)	164.2 ± 2.6 (6)	ns	0.001	ns
FEMUR	196.5 ± 3.3 (5)	198.8 ± 1.2 (6)	208.5 ± 2.4 (9)	201.3 ± 4.1 (12)	219.1 ± 2.6 (5)	213.7 ± 4.0 (6)	ns	0.001	ns

*Mean ± SEM for the number of animals indicated in parentheses for control (CONTROL) and immobilized (IMMO) groups are shown for every period. Bone Mineral density (BMD) was measured by peripheral Dual-energy X-ray Absorptiometry (pDEXA) analysis and expressed as mg/cm^2^. Statistical significance was tested by a full factorial two-way ANOVA (fixed factors: I, immobilization, T, Time; IxT denotes the interaction term). p-values are shown in the three columns on the right (ns, non-significant differences, which implies a p-value equal to or greater than 0.05)*.

## Discussion

It is known that a period of bed rest leads to physical inactivity status with an associated reduction of energy requirements and appetite. Consequently, food intake generally declines, resulting in an inadequate dietary protein consumption to allow proper muscle mass maintenance (Wall and van Loon, 2013). This is also observed in our study. It could be speculated that the feeding behavior observed in cage-restricted rats could reflect the environmental stress at which apparently the animals were submitted (Zylan and Brown, 1996). Indeed, the inhibition of vegetative functions, such as appetite and feeding, is considered an acute physiological response resulting from the effects of stress, induced by the immobilization device, on the appetite-satiety centers in the hypothalamus (Shimizu et al., 1989; Krahn et al., 1990; Charmandari et al., 2005). Certain peptides and neurotransmitters are involved in this response, such as monoamines (Kennett et al., 1987) corticotrophin-releasing hormone (CRH) (Krahn et al., 1988, 1990; Rich, 2005) and others (Charmandari et al., 2005). The body's response to a stressful stimulus is regulated by the hypothalamic-pituitary-adrenal (HPA) axis through hormonal feedback (Cruthirds et al., 2011). The HPA axis involves the release of corticotrophin-releasing hormone (CTH) from the hypothalamus, which modulates the secretion of adrenocorticotropin hormone (ACTH) from the anterior pituitary, which then controls the secretion of glucocorticoid from the adrenal glands (Cruthirds et al., 2011). Many immobilization models in rodents are defined as severe chronic stressors (Martí et al., 1994). Ricart-Jané et al. reported that immobilization resulted in decreased body weight gain and food intake, together with an increase in the weight of the adrenal glands. It also resulted in a decrease in liver glycogen, all of them signs of chronic stress (Ricart-Jané et al., 2002). However, the results obtained concerning corticosterone concentrations in our immobilization model confirmed that the changes observed in food intake could not be attributed to stress. In fact, the circulating corticosteroids concentrations together with the unchanged adrenal glands weight (Table 2) and the unaltered body weight gain (Table 1), confirmed that the immobilization model proposed does not represent a physical stressor for the animal.

No changes were observed on glucose metabolism when the immobilization period was longer than 7 days, possibly as a consequence of the activation of a compensatory response improving the ability of the organism to adapt and increase its chance for the survival. However, further analysis is mandatory to clarify the molecular pathways underlying the hyperglycaemia observed.

**Figure 4:**
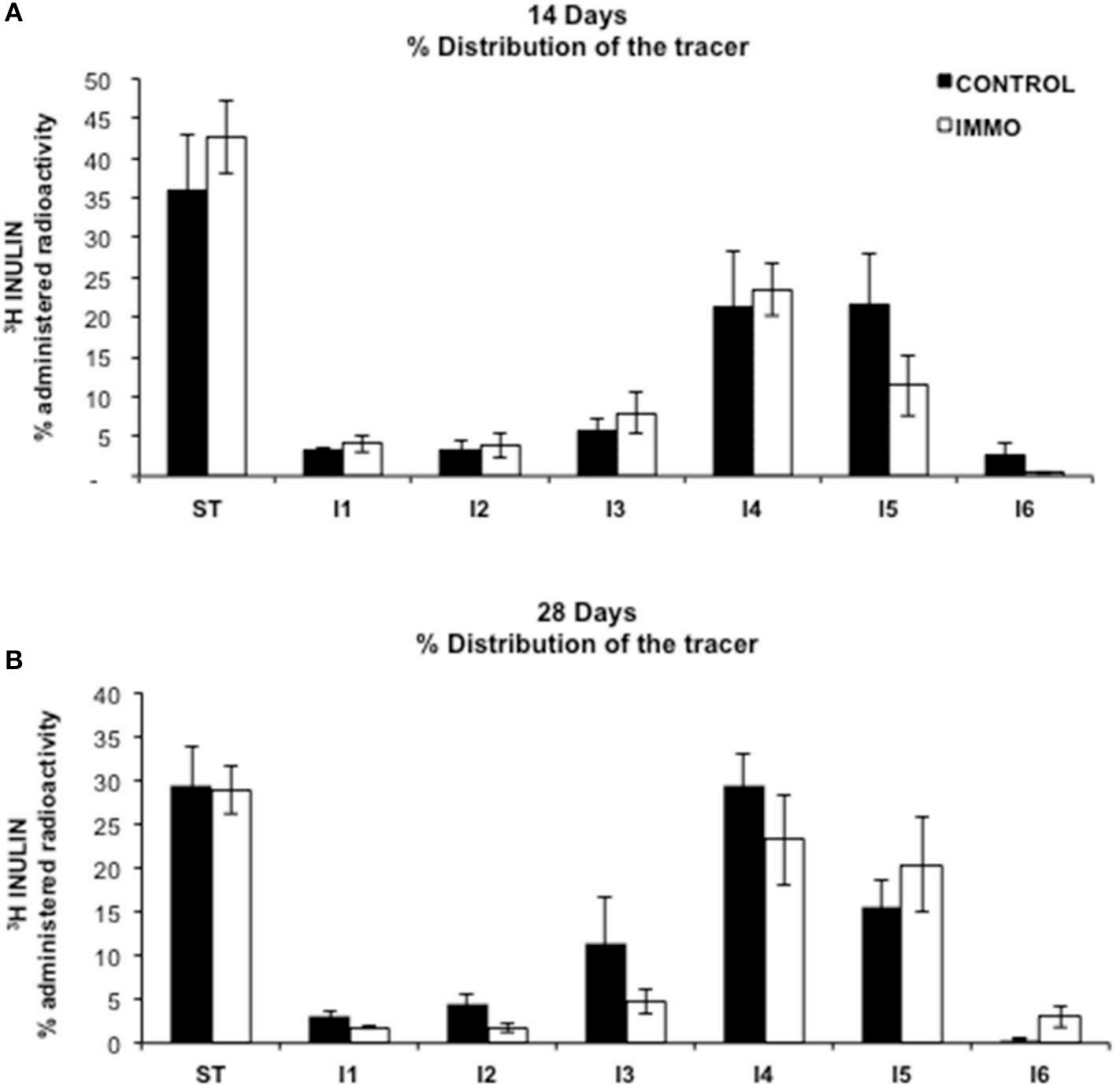
**Gastrointestinal motility in immobilized Wistar rats**. The represented results are mean values ± SEM for control group (CONTROL) (*n* = 5 animals) and immobilized group (IMMO) (*n* = 6) animals in each gastrointestinal segment. Gastrointestinal distribution of orally-administrated [^3^H]Inulin. ST: Stomach, I1 to I6: Intestine equivalent segments from duodenal to colon. Data were analyzed by a Linear Mixed Model, being time (T) and immobilization (I) two crossed between-subjects factors, and gastrointestinal segment (G) the within-subjects factor (repeated measures). Restricted Maximum Likelihood (REML) method was used to fit the model. According to Akaike Information Criterion (AIC) and Schwarz Bayesian Information Criterion (BIC), a heterogeneous Toeplitz covariance structure was finally chosen. Only significant differences among the gastrointestinal segments were detected (G, *p* = 0.000).

Several studies indicate an inverse relationship between physical activity and risk of gastrointestinal-related disease, such as colon cancer, diverticular disease, cholelithiasis or constipation (Everhart et al., 1989; Aldoori et al., 1995; Colditz et al., 1997; Leitzmann et al., 1998, 1999; Peters et al., 2001). In particular, the last is an uncomfortable gastrointestinal disturb, very common in the Western society, that is strictly associated with diet and physical exercise and characterized by hard stool consistency, straining and incomplete defecation (Sandler and Drossman, 1987; Dukas et al., 2003). For this reasons, we evaluated the effects of reduced physical mobility induced by our model on bowel functionality of the immobilized rats using a methodology based on the gastrointestinal distribution of [^3^H]inulin, an indigestible carbohydrate (Arbós et al., 1993). From our data, physical inactivity did not influence gastrointestinal motility (Figure 4).

**Figure 5:**
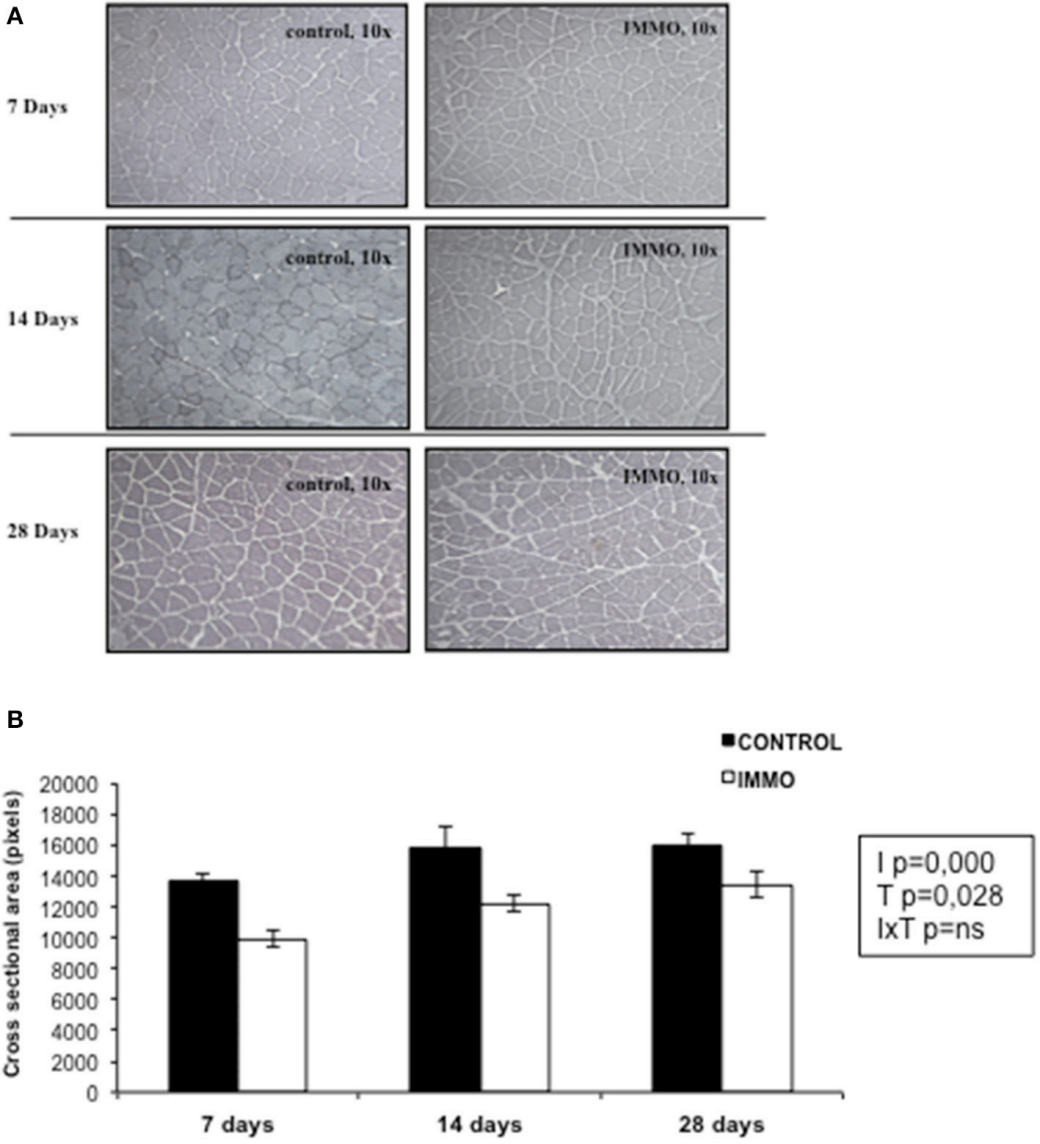
**Muscle fiber size in immobilized Wistar rats. (A)** Representative images of muscle tissue sections stained with haematoxylin and eosin. **(B)** Muscle fiber cross-sectional area (pixels) of soleus muscle was determined on randomly chosen 100 individual fibers per animal by the Matic Image Plus 2. Bars and segments represents the mean values ± SEM for each group: day 7: CONTROL (*n* = 3) and immobilized group (IMMO) (*n* = 5); day 14: CONTROL (*n* = 6) and immobilized group (IMMO) (*n* = 11); day 28: CONTROL (*n* = 4) and immobilized group (IMMO) (*n* = 5). Statistical significance of the results were assessed by a full factorial two-way ANOVA (fixed factors: I = immobilization, T = Time; IxT denotes the interaction term): I, *p* = 0.000; T, *p* = 0.028; IxT, *p* = ns.

In patients, a period of prolonged bed rest, ranging between 10 and 42 days, is accompanied by a variable loss in muscle strength (between 0.3 and 4.2% per day) (Wall et al., 2013). In our study, immobilization resulted in a significant decrease in soleus weight (Table 3). This result is in accordance with that observed in many other rodent models of immobilization and hindlimb unloading, which have reported a greater muscle loss in the extensor muscles of the ankle (i.e., soleus and gastrocnemius) rather than the flexor muscles (i.e., tibialis anterior and *extensor digitorum longus*) (Thomason and Booth, 1990; Ohira et al., 2002; Adams et al., 2003; Zhong et al., 2005), demonstrating a preferential sensitivity to unloading of muscles predominately expressing the slow MHC phenotype (Baldwin et al., 2013). In addition of the decreased soleus weight, a significant decrease in the cross sectional area (CSA) of the muscle fibers was observed in all the immobilization groups studied (Figure 5). Indeed, a reduced CSA is the main characteristic morphological alteration resulting from muscle atrophy. Other alterations are: sarcomere dissolution, endothelial degradation, build-up of connective tissue between muscle fibers, reduction in the number of mitochondria, and a reduction in capillary density (Tyml et al., 1990; Ohira et al., 2002; Nielsen et al., 2010; Giordano et al., 2014). Our data are in agreement with the reduced CSA value reported in muscle fibers after 16 days of hindlimb suspension (Ishihara et al., 2002), and after 7 days of hindlimb casting immobilization (Talbert et al., 2013). Our model is characterized by a slower rate of muscle wasting in comparison with other immobilization models. For instance, 7 and 14 days of hindlimb suspension induce around 20 and 50%, respectively, of atrophy in the soleus muscle (Isfort et al., 2002; De Boer et al., 2007). Meanwhile, 8 days of hindlimb immobilization using the plaster-cast model causes an atrophy of 23% in the soleus muscle (Vazeille et al., 2008). Other studies, using animal models, have suggested that the atrophy-induced disuse is driven by a decreased rate of protein synthesis and an increased rate of protein degradation (Booth and Seider, 1979; Tucker et al., 1981; Medina et al., 1995; Taillandier et al., 1996; Krawiec et al., 2005). Thomas on and Booth proposed a model to describe the mechanisms responsible for muscle loss observed in rat soleus muscle following hindlimb unloading. They identify a very fast decrease in protein synthesis rate followed by a gradually increase of the protein degradation rate which reached a peak by 15 days and then declined to below baseline levels (Thomason and Booth, 1990). Conversely, in humans subjects, the muscle atrophy observed during prolonged muscle disuse (>10 days) is a direct consequence of a reduced post-absorptive and post-prandial muscle synthesis rather than due to changes in muscle protein breakdown rates (Wall et al., 2013). Meanwhile during short term disuse (<10 days), the rapid muscle loss is probably due to increased muscle protein degradation that takes place simultaneously to reduced muscle protein synthesis (Wall et al., 2013). Moreover, and in accordance to human studies (Seki et al., 2001), the data presented in Table 3 showed significant decrease of muscle force after 7 and 14 days of physical inactivity therefore validating further the efficacy of the model to mimic the physiological effects of bed rest in humans.

Heart weight was also decreased after 7 and 14 days of immobilization (Table 3). This result is in the agreement with published data obtained in humans showing that 6 weeks of horizontal bed rest cause cardiac atrophy (8%) as a consequence of physiological adaptation to a reduced maximal oxygen uptake and to reserve capacity in perform physical work (Convertino, 1997; Perhonen et al., 2001). On the same lines, Evans and Ivy using experimental animal models, underlined the ability of the hindlimb immobilization technique to induce a generalized catabolic state that was not only restricted to the muscle immobilized but also affected the cardiac tissue, impairing its aerobic capacity and reducing muscle size (Evans and Ivy, 1982).

Several studies have shown that disuse atrophy is associated with bone loss (Bloomfield, 1997; Collet et al., 1997; Kiratli et al., 2000). The bone mineral density (BMD) is the result of a dynamic process, called remodeling, which involves the removal of old or damaged bone by osteoclasts (bone resorption) and the subsequent replacement of new bone formed by osteoblasts (bone formation) (Feng and McDonald, 2011). The absence of intermittent mechanical solicitations, usually produced during loading and muscle contractions, is responsible of progressive deformations of cartilages and alterations of bone remodeling which results in a disorder termed immobilization-induced osteoporosis (Feng and McDonald, 2011). Indeed, studies in healthy subjects have shown that only 24 h of immobilization induced a rise in the osteoclast activity associated with a pronounced increase of bone resorption markers (Suzuki et al., 1994; Heer et al., 2005). In our model this loss of bone density was also observed. In other models, it has been shown that 10 days of hindlimb immobilization by plaster cast caused bone loss as measured by a reduced bone mineral density of the femur (−9%) and a decreased trabecular bone volumen of the tibial metaphysis (−25%) (Hott et al., 2003). The loss of bone weight induced by casting immobilization was mainly due to mineral losses -as indicated by changes in wet weight, ash weight, and calcium content- with a substantial part of the decrease affecting the trabecular bone and not the reduction of external bone volume (Tuukkanen et al., 1991).

## Conclusions

The muscle atrophy together with a significant decline in muscle mass and force, and the loss of bone mass, define the proposed immobilization model as a new tool for the study of disuse-muscle atrophy. Our new procedure overcomes several limitations of the other commonly used immobilization ones, with the advantages of being a low-cost and non-invasive model, standardized, reproducible and easy to implement. It does not require specific expensive equipment, and it maintains neural innervation to the musculature, while it does not alter the body weight and permits recovery-type studies to be performed with a low level of stress. Furthermore, in terms of lifespan, the immobilization periods used here are far longer than those used previously with human studies, characterizing therefore a “soft,” “slow” and long-term model of muscle atrophy that better reproduces the muscle loss of a patient in bed rest condition. The ability of the rat to slightly move in circles inside the restraint cage reflects the real-life circumstances of patients that are not completely immobilized.

Altogether, the results presented here propose a new model for studying the effects of bed rest in experimental animals by reducing cage volume. In this model the number of movements -particularly locomotion ones- are virtually abolished, in a similar situation as is found during bed rest. The muscle atrophy, a significant decline in muscle force together with the loss of bone mass are the main effects of the proposed immobilization model which may potentially serve to investigate the effects of bed-rest in pathological states characterized by a catabolic condition, such as diabetes or cancer.

## Author contributions

Each author has participated sufficiently, intellectually or practically, in the work to take public responsibility for the content of the article, including the conception, design, and conduction of the experiment and for data interpretation (authorship). EM and SB carried out the studies, sample analysis, data analyses, performed the statistical analysis and helped to draft the manuscript. FO helped to realize the statistical analysis and to draft the manuscript. MT, MR, MB, VG helped to carry out the studies, sample analysis and data analysis. JA, JL, MM, RR provide the intellectual input and designs and approves the protocols to be followed in the study. JA, FL, JL, MM, RR conceived the study, participated in the design, coordination of the study, drafted the manuscript and revised it critically.

### Conflict of interest statement

The authors declare that the research was conducted in the absence of any commercial or financial relationships that could be construed as a potential conflict of interest.

## References

[B1] Abramoff M. D. Magalhães P. J. Ram S. J. (2004). Image processing with ImageJ. Biophotonics Int. 11, 36–42.

[B2] Adams G. R. Caiozzo V. J. Baldwin K. M. (2003). Skeletal muscle unweighting: spaceflight and ground-based models. J. Appl. Physiol. 95, 2185–2201. 10.1152/japplphysiol.00346.2003 14600160

[B3] Aldoori W. H. Giovannucci E. L. Rimm E. B. Ascherio A. Stampfer M. J. Colditz G. A.. (1995). Prospective study of physical activity and the risk of symptomatic diverticular disease in men. Gut 36, 276–282. 10.1136/gut.36.2.276 7883230 PMC1382417

[B4] Arbós J. Zegrí A. López-Soriano F. J. Argilés J. M. (1993). A simple method for determining the rate of gastrointestinal transit in the rat. Arch. Int. hysiol. Biochim. Biophys. 101, 113–115. 10.3109/13813459309008878 7689354

[B5] Baldwin K. M. Haddad F. Pandorf C. E. Roy R. R. Edgerton V. R. (2013). Alterations in muscle mass and contractile phenotype in response to unloading models: role of transcriptional/pretranslational mechanisms. Front. Physiol. 4:284. 10.3389/fphys.2013.00284 24130531 PMC3795307

[B6] Bialek P. Morris C. Parkington J. St Andre M. Owens J. Yaworsky P.. (2011). Distinct protein degradation profiles are induced by different disuse models of skeletal muscle atrophy. Physiol. Genomics 43, 1075–1086. 10.1152/physiolgenomics.00247.2010 21791639 PMC3217324

[B7] Bloomfield S. A. (1997). Changes in musculoskeletal structure and function with prolonged bed rest. Med. Sci. Sports Exerc. 29, 197–206. 10.1097/00005768-199702000-00006 9044223

[B8] Booth F. W. Seider M. J. (1979). Early change in skeletal muscle protein synthesis after limb immobilization of rats. J. Appl. Physiol. 47, 974–977. 511723 10.1152/jappl.1979.47.5.974

[B9] Buynitsky T. Mostofsky D. I. (2009). Restraint stress in biobehavioral research: recent developments. Neurosci. Biobehav. Rev. 33, 1089–1098. 10.1016/j.neubiorev.2009.05.004 19463853

[B10] Charmandari E. Tsigos C. Chrousos G. (2005). Endocrinology of the stress response. Annu. Rev. Physiol. 67, 259–284. 10.1146/annurev.physiol.67.040403.120816 15709959

[B11] Chopard A. Hillock S. Jasmin B. J. (2009). Molecular events and signalling pathways involved in skeletal muscle disuse-induced atrophy and the impact of countermeasures. J. Cell. Mol. Med. 13, 3032–3050. 10.1111/j.1582-4934.2009.00864.x 19656243 PMC4516463

[B12] Colditz G. A. Cannuscio C. C. Frazier A. L. (1997). Physical activity and reduced risk of colon cancer: implications for prevention. Cancer Causes Control 8, 649–667. 10.1023/A:1018458700185 9242482

[B13] Collet P. Uebelhart D. Vico L. Moro L. Hartmann D. Roth M.. (1997). Effects of 1- and 6-month spaceflight on bone mass and biochemistry in two humans. Bone 20, 547–551. 10.1016/S8756-3282(97)00052-5 9177869

[B14] Convertino V. A. (1997). Cardiovascular consequences of bed rest: effect on maximal oxygen uptake. Med. Sci. Sports Exerc. 29, 191–196. 10.1097/00005768-199702000-00005 9044222

[B15] Corcoran P. J. (1991). Use it or lose it–the hazards of bed rest and inactivity. *West. J*. Med. 154, 536–538. 1866946 PMC1002823

[B16] Cruthirds D. F. Siangco A. L. Hartman C. J. Sandefur D. C. Spencer J. M. Dyer C. A.. (2011). Effects of immobilization stress and hormonal treatment on nociception. AANA J. 79, 375–380. 23256266

[B17] De Boer M. D. Maganaris C. N. Seynnes O. R. Rennie M. J. Narici M. V. (2007). Time course of muscular, neural and tendinous adaptations to 23 day unilateral lower-limb suspension in young men. J. Physiol. 583, 1079–1091. 10.1113/jphysiol.2007.135392 17656438 PMC2277190

[B18] Dittmer D. K. Teasell R. (1993). Complications of immobilization and bed rest. Part 1: musculoskeletal and cardiovascular complications. Can. Fam. Physician 39, 1428–1432, 1435–1437. 8324411 PMC2379624

[B19] Dukas L. Willett W. C. Giovannucci E. L. (2003). Association between physical activity, fiber intake, and other lifestyle variables and constipation in a study of women. Am. J. Gastroenterol. 98, 1790–1796. 10.1111/j.1572-0241.2003.07591.x 12907334

[B20] Evans W. J. Ivy J. L. (1982). Effects of testosterone propionate on hindlimb-immobilized rats. J. Appl. Physiol. 52, 1643–1647. 10.1249/00005768-198202000-00043 7107474

[B21] Everhart J. E. Go V. L. Johannes R. S. Fitzsimmons S. C. Roth H. P. White L. R. (1989). A longitudinal survey of self-reported bowel habits in the United States. Dig. Dis. Sci. 34, 1153–1162. 10.1007/BF01537261 2787735

[B22] Feng X. McDonald J. M. (2011). Disorders of bone remodeling. Annu. Rev. Pathol. 6, 121–145. 10.1146/annurev-pathol-011110-130203 20936937 PMC3571087

[B23] Frimel T. N. Kapadia F. Gaidosh G. S. Li Y. Walter G. A. Vandenborne K. (2005). A model of muscle atrophy using cast immobilization in mice. Muscle Nerve 32, 672–674. 10.1002/mus.20399 16025524

[B24] Giordano F. M. Vizziello E. Tidball J. G. Falcieri E. Curzi D. (2014). Plantaris muscle adaptation to atrophy generated by disuse: an ultrastructural study. Microscopie 11, 31–36. 10.4081/microscopie.2014.4992

[B25] Griffin M. G. Kimble R. Hopfer W. Pacifici R. (1993). Dual-energy x-ray absorptiometry of the rat: accuracy, precision, and measurement of bone loss. J. Bone Miner. Res. 8, 795–800. 10.1002/jbmr.5650080704 8352062

[B26] Hauger R. L. Millan M. A. Lorang M. Harwood J. P. Aguilera G. (1988). Corticotropin-releasing factor receptors and pituitary adrenal responses during immobilization stress. Endocrinology 123, 396–405. 10.1210/endo-123-1-396 2838259

[B27] Heer M. Baecker N. Mika C. Boese A. Gerzer R. (2005). Immobilization induces a very rapid increase in osteoclast activity. Acta Astronaut. 57, 31–36. 10.1016/j.actaastro.2004.12.007 15900645

[B28] Hott M. Deloffre P. Tsouderos Y. Marie P. J. (2003). S12911-2 reduces bone loss induced by short-term immobilization in rats. Bone 33, 115–123. 10.1016/S8756-3282(03)00115-7 12919706

[B29] Isfort R. J. Wang F. Greis K. D. Sun Y. Keough T. W. Farrar R. P.. (2002). Proteomic analysis of rat soleus muscle undergoing hindlimb suspension-induced atrophy and reweighting hypertrophy. Proteomics 2, 543–550. 10.1002/1615-9861(200205)2:5<543::AID-PROT543>3.0.CO;2-K 11987128

[B30] Ishihara A. Nishikawa W. Kawano F. Fukunaga K. Ohira Y. (2002). Effects of hindlimb suspension on soleus muscle fibers and their spinal motoneurons in Wistar Hannover rats. J. Gravit. Physiol. 9, P141–P142. 15002520

[B31] Jackman R. W. Kandarian S. C. (2004). The molecular basis of skeletal muscle atrophy. Am. J. Physiol. Cell Physiol. 287, C834–C843. 10.1152/ajpcell.00579.2003 15355854

[B32] Kennett G. A. Dourish C. T. Curzon G. (1987). 5-HT1B agonists induce anorexia at a postsynaptic site. Eur. J. Pharmacol. 141, 429–435. 10.1016/0014-2999(87)90561-9 3666036

[B33] Kiratli B. J. Smith A. E. Nauenberg T. Kallfelz C. F. Perkash I. (2000). Bone mineral and geometric changes through the femur with immobilization due to spinal cord injury. J. Rehabil. Res. Dev. 37, 225–233. 10850829

[B34] Krahn D. D. Gosnell B. A. Levine A. S. Morley J. E. (1988). Behavioral effects of corticotropin-releasing factor: localization and characterization of central effects. Brain Res. 443, 63–69. 10.1016/0006-8993(88)91598-3 2834018

[B35] Krahn D. D. Gosnell B. A. Majchrzak M. J. (1990). The anorectic effects of CRH and restraint stress decrease with repeated exposures. Biol. Psychiatry 27, 1094–1102. 10.1016/0006-3223(90)90046-5 2340320

[B36] Krawiec B. J. Frost R. A. Vary T. C. Jefferson L. S. Lang C. H. (2005). Hindlimb casting decreases muscle mass in part by proteasome-dependent proteolysis but independent of protein synthesis. Am. J. Physiol. Endocrinol. Metab. 289, E969–E980. 10.1152/ajpendo.00126.2005 16046454

[B37] Leitzmann M. F. Giovannucci E. L. Rimm E. B. Stampfer M. J. Spiegelman D. Wing A. L.. (1998). The relation of physical activity to risk for symptomatic gallstone disease in men. Ann. Intern. Med. 128, 417–425. 10.7326/0003-4819-128-6-199803150-00001 9499324

[B38] Leitzmann M. F. Rimm E. B. Willett W. C. Spiegelman D. Grodstein F. Stampfer M. J.. (1999). Recreational physical activity and the risk of cholecystectomy in women. N. Engl. J. Med. 341, 777–784. 10.1056/NEJM199909093411101 10477775

[B39] Machida S. Booth F. W. (2004). Regrowth of skeletal muscle atrophied from inactivity. Med. Sci. Sports Exerc. 36, 52–59. 10.1249/01.MSS.0000106175.24978.84 14707768

[B40] Martí O. Martí J. Armario A. (1994). Effects of chronic stress on food intake in rats: influence of stressor intensity and duration of daily exposure. Physiol. Behav. 55, 747–753. 10.1016/0031-9384(94)90055-8 8190805

[B41] Medina R. Wing S. S. Goldberg A. L. (1995). Increase in levels of polyubiquitin and proteasome mRNA in skeletal muscle during starvation and denervation atrophy. Biochem. J. 307(Pt 3), 631–637. 10.1042/bj3070631 7741690 PMC1136697

[B42] Midrio M. (2006). The denervated muscle: facts and hypotheses. A historical review. Eur. J. Appl. Physiol. 98, 1–21. 10.1007/s00421-006-0256-z 16896733

[B43] Morey-Holton E. R. Globus R. K. (2002). Hindlimb unloading rodent model: technical aspects. J. Appl. Physiol. 92, 1367–1377. 10.1152/japplphysiol.00969.2001 11895999

[B44] Musacchia X. J. Steffen J. M. Fell R. D. (1988). Disuse atrophy of skeletal muscle: animal models. Exerc. Sport Sci. Rev. 16, 61–87. 10.1249/00003677-198800160-00005 3292267

[B45] Nielsen J. Suetta C. Hvid L. G. Schrøder H. D. Aagaard P. Ortenblad N. (2010). Subcellular localization-dependent decrements in skeletal muscle glycogen and mitochondria content following short-term disuse in young and old men. Am. J. Physiol. Endocrinol. Metab. 299, E1053–E1060. 10.1152/ajpendo.00324.2010 20858747

[B46] Nixon J. P. Zhang M. Wang C. Kuskowski M. A. Novak C. M. Levine J. A.. (2010). Evaluation of a quantitative magnetic resonance imaging system for whole body composition analysis in rodents. Obesity 18, 1652–1659. 10.1038/oby.2009.471 20057373 PMC2919581

[B47] Ohira Y. Yoshinaga T. Nomura T. Kawano F. Ishihara A. Nonaka I.. (2002). Gravitational unloading effects on muscle fiber size, phenotype and myonuclear number. Adv. Space Res. 30, 777–781. 10.1016/S0273-1177(02)00395-2 12530363

[B48] Perhonen M. A. Franco F. Lane L. D. Buckey J. C. Blomqvist C. G. Zerwekh J. E.. (2001). Cardiac atrophy after bed rest and spaceflight. J. Appl. Physiol. 91, 645–653. 11457776 10.1152/jappl.2001.91.2.645

[B49] Peters H. P. De Vries W. R. Vanberge-Henegouwen G. P. Akkermans L. M. (2001). Potential benefits and hazards of physical activity and exercise on the gastrointestinal tract. Gut 48, 435–439. 10.1136/gut.48.3.435 11171839 PMC1760153

[B50] Reeves P. G. Nielsen F. H. Fahey G. C. (1993). AIN-93 purified diets for laboratory rodents: final report of the American Institute of Nutrition *ad hoc* writing committee on the reformulation of the AIN-76A rodent diet. J. Nutr. 123, 1939–1951. 8229312 10.1093/jn/123.11.1939

[B51] Ricart-Jané D. Rodríguez-Sureda V. Benavides A. Peinado-Onsurbe J. López-Tejero M. D. Llobera M. (2002). Immobilization stress alters intermediate metabolism and circulating lipoproteins in the rat. Metabolism 51, 925–931. 10.1053/meta.2002.33353 12077743

[B52] Rich E. L. (2005). Exposure to chronic stress downregulates corticosterone responses to acute stressors. AJP Regul. Integr. Comp. Physiol. 288, R1628–R1636. 10.1152/ajpregu.00484.2004 15886358

[B53] Sandler R. S. Drossman D. A. (1987). Bowel habits in young adults not seeking health care. Dig. Dis. Sci. 32, 841–845. 10.1007/BF01296706 3608732

[B54] Seki K. Taniguchi Y. Narusawa M. (2001). Effects of joint immobilization on firing rate modulation of human motor units. J. Physiol. 530, 507–519. 10.1111/j.1469-7793.2001.0507k.x 11158280 PMC2278422

[B55] Shimizu N. Oomura Y. Kai Y. (1989). Stress-induced anorexia in rats mediated by serotonergic mechanisms in the hypothalamus. Physiol. Behav. 46, 835–841. 10.1016/0031-9384(89)90045-0 2628995

[B56] Smith C. (2012). Using rodent models to simulate stress of physiologically relevant severity: when, why and how, in Glucocorticoids - New Recognition of Our Familiar Friend, ed Qian X. (InTech), 211–230.

[B57] Suzuki Y. Murakami T. Haruna Y. Kawakubo K. Goto S. Makita Y.. (1994). Effects of 10 and 20 days bed rest on leg muscle mass and strength in young subjects. Acta Physiol. Scand. Suppl. 616, 5–18. 8042525

[B58] Taillandier D. Aurousseau E. Meynial-Denis D. Bechet D. Ferrara M. Cottin P.. (1996). Coordinate activation of lysosomal, Ca 2+-activated and ATP-ubiquitin-dependent proteinases in the unweighted rat soleus muscle. Biochem. J. 316(Pt 1), 65–72. 10.1042/bj3160065 8645234 PMC1217351

[B59] Talbert E. E. Smuder A. J. Min K. Kwon O. S. Szeto H. H. Powers S. K. (2013). Immobilization-induced activation of key proteolytic systems in skeletal muscles is prevented by a mitochondria-targeted antioxidant. J. Appl. Physiol. 115, 529–538. 10.1152/japplphysiol.00471.2013 23766499

[B60] Thomason D. B. Booth F. W. (1990). Atrophy of the soleus muscle by hindlimb unweighting. J. Appl. Physiol. 68, 1–12. 2179205 10.1152/jappl.1990.68.1.1

[B61] Toledo M. Busquets S. Sirisi S. Serpe R. Orpí M. Coutinho J.. (2011). Cancer cachexia: physical activity and muscle force in tumour-bearing rats. Oncol. Rep. 25, 189–193. 10.3892/or_00001060 21109976

[B62] Tucker K. R. Seider M. J. Booth F. W. (1981). Protein synthesis rates in atrophied gastrocnemius muscles after limb immobilization. J. Appl. Physiol. 51, 73–77. 7263427 10.1152/jappl.1981.51.1.73

[B63] Tuukkanen J. Wallmark B. Jalovaara P. Takala T. Sjögren S. Väänänen K. (1991). Changes induced in growing rat bone by immobilization and remobilization. Bone 12, 113–118. 10.1016/8756-3282(91)90009-8 2064838

[B64] Tyml K. Mathieu-Costello O. Budreau C. H. (1990). Microvascular response to ischemia, and tissue structure, in normal and atrophied skeletal muscle. Microvasc. Res. 39, 223–239. 10.1016/0026-2862(90)90072-Y 2352492

[B65] Vazeille E. Codran A. Claustre A. Averous J. Listrat A. Béchet D.. (2008). The ubiquitin-proteasome and the mitochondria-associated apoptotic pathways are sequentially downregulated during recovery after immobilization-induced muscle atrophy. Am. J. Physiol. Endocrinol. Metab. 295, E1181–E1190. 10.1152/ajpendo.90532.2008 18812460

[B66] Wall B. T. Dirks M. L. van Loon L. J. C. (2013). Skeletal muscle atrophy during short-term disuse: implications for age-related sarcopenia. Ageing Res. Rev. 12, 898–906. 10.1016/j.arr.2013.07.003 23948422

[B67] Wall B. T. van Loon L. J. C. (2013). Nutritional strategies to attenuate muscle disuse atrophy. Nutr. Rev. 71, 195–208. 10.1111/nure.12019 23550781

[B68] Wood G. E. Young L. T. Reagan L. P. McEwen B. S. (2003). Acute and chronic restraint stress alter the incidence of social conflict in male rats. Horm. Behav. 43, 205–213. 10.1016/S0018-506X(02)00026-0 12614651

[B69] Zhong H. Roy R. R. Siengthai B. Edgerton V. R. (2005). Effects of inactivity on fiber size and myonuclear number in rat soleus muscle. J. Appl. Physiol. 99, 1494–1499. 10.1152/japplphysiol.00394.2005 15994244

[B70] Zylan K. D. Brown S. D. (1996). Effect of stress and food variety on food intake in male and female rats. Physiol. Behav. 59, 165–169. 10.1016/0031-9384(95)02039-X 8848477

